# Microbial contamination and tissue procurement location: A conventional operating room is not mandatory. An observational study

**DOI:** 10.1371/journal.pone.0210140

**Published:** 2019-01-08

**Authors:** Benjamin Louart, Claire Charles, Tri-Long Nguyen, Nicolas Builles, Claire Roger, Jean-Yves Lefrant, Florence Vachiery-Lahaye, John De Vos, Guilhem Couderc, Laurent Muller

**Affiliations:** 1 Department of Anesthesiology Intensive Care, Pain and Emergency medicine, Nîmes University Hospital, Montpellier University, Nîmes, France; 2 Department of Clinical Pharmacy, Niîmes University Hospital, Niîmes, France; 3 Banque de Tissue, Centre des Collections Biologiques Hospitalières de Montpellier, Centre Hospitalier Universitaire de Montpellier, Montpellier, France; 4 Coordination Hospitalière des Prélèvements d'Organes et de Tissus, Centre Hospitalier Universitaire de Montpellier, Montpellier, France; Policlinico S. Orsola-Malpighi, ITALY

## Abstract

**Background:**

Standard operating rooms (SOR) are assumed to be the best place to prevent microbial contamination when performing tissue procurement. However, mobilizing an operating room is time and cost consuming if no organ retrieval is performed. In such case, non-operating dedicated rooms (NODR) are usually recommended by European guidelines for tissue harvesting. Performing the tissue retrieval in the Intensive care unit (ICU) when possible might be considered as it allows a faster and simpler procedure.

**Objective:**

Our primary objective was to study the relationship between the risk of microbial contamination and the location (ICU, SOR or NODR) of the tissue retrieval in heart-beating and non-heart-beating deceased donors.

**Materials and method:**

We retrospectively reviewed all deceased donors’ files of the local tissue banks of Montpellier and Marseille from January 2007 to December 2014. The primary endpoint was the microbial contamination of the grafts. We built a multivariate regression model and used a GEE (generalized estimating equations) allowing us to take into account the clustered structure of our data.

**Results:**

2535 cases were analyzed involving 1027 donors. The retrieval took place for 1189 in a SOR, for 996 in a hospital mortuary (NODR) and for 350 in an ICU. 285 (11%) microbial contaminations were revealed. The multivariate analysis found that the location in a hospital mortuary was associated with a lower risk of contamination (OR 0.43, 95% CI [0.2–0.91], p = 0.03). A procurement performed in the ICU was not associated with a significant increased risk (OR 0.62, 95% CI [0.26–1.48], p = 0.4).

**Conclusion:**

According to our results, performing tissue procurement in dedicated non-sterile rooms could decrease the rate of allograft tissue contamination. This study also suggests that in daily clinical practice, transferring patients from ICU to SOR for tissue procurement could be avoided as it does not lead to less microbial contamination.

## Introduction

Tissue graft is a life enhancing and occasionally a life saving therapy[1]. It remains the ultimate treatment of various diseases and transplantation of tissues can range from life-saving treatments (e.g. of catastrophic burns) to quality-of-life improvements. More than 1.5 millions tissue grafts are performed annually in USA[2], and approximately 38 000 per year in France[3]. The continuous improvement and actualization of international guidelines have led to increase the quality and safety of tissue graft over the last 20 years[4]. The majority (80%) of tissue allografts are bone femoral heads, obtained from living donors during hip surgery[3]. Hip surgery is a highly sterile surgical procedure, limiting the risk of microbial contamination. About 20% tissue grafts are performed from deceased donors (heart-beating and non-heart-beating deceased donors), by descending order: corneal, skin, artery, cardiac valves and full-length skeletal bones grafts [1, 3]. In deceased donors, because of the natural exposure of skin and cornea to bacteria, tissue retrieval procedures are at high risk of microbiological contamination[1, 2]. This risk has been widely reported. The main microorganisms isolated on tissue samples obtained from deceased donors (heart beating or non-heart beating) are *Staphylococcus spp*, *Streptococci*, *Propionibacterium*, *Clostridium*, *Escherichia coli* and Fungi, mainly *Candida species[5–9]*. Several risks factors of microbiological contamination related to donors have been identified: duration between death and retrieval, donor age, cause of death. However, the respective importance of such factors remains debated [6, 10–13].

The EDQM guidelines (European Directorate for the Quality of Medicines and Healthcare) recommend that tissue collection should be performed as soon as possible after circulatory arrest, ideally in the 24 following hours if body refrigeration is achieved in the 6 hours following death[4]. Theoretically, standard operating room (SOR) represents the best place to perform tissue collection in terms of infectious risk. Indeed, operating theaters are clean controlled and air quality monitored area with a classification ISO7 thanks to the International organization for standardization standards and to French guidelines [14, 15]. However, mobilizing an operating room is time and cost consuming if no organ retrieval is performed. The third EDQM guidelines [4] describe 4 categories of facilities for procurement: operating theater or equivalent (corresponding to SOR), dedicated procurement area with controlled cleaning and with or without routine air-quality monitoring (corresponding to Non-operating dedicated room, NODR) and finally non-dedicated area with only local cleaning. The choice of the location may depend on the contamination risk assessment. Factors are provided to help decision, but no clear rule is given and this risk assessment is subjective and could lead to different appreciations. Thanks to these guidelines, for skin and corneal grafts, SOR is not necessary, while for cardiovascular tissues SOR is recommended but not mandatory[4]. Because a significant proportion of patients die in Intensive care unit (ICU), the possibility of performing tissue retrieval in ICU just after death might be considered as it would allow a faster procedure. Moreover, an ICU room has theoretically the same characteristics as a NODR. Interestingly, the influence of the type of room dedicated to tissue retrieval in terms of tissue microbiological contamination has never been studied. Therefore, it appears licit to compare for deceased donors the rate of microbiological contamination according to the location of the tissue collection.

## Materials and methods

### Study design

After Institutional Review Board approval (N°15/06-12), all deceased (brain death and circulatory death) donors’ files of the local tissue banks of Montpellier and Marseille from January 2007 to December 2014 were retrospectively reviewed. All data we worked on from donors are anonymously recorded in tissue establishments.

### Ethical issues

In France, the legal consent system for expressing consent to donation is an opting-out system, as in the majority of European countries (4), in which consent to donation is presumed where no objection to donation has been registered by an individual during their lifetime or is known to the donor's family[16]. The procurement staff discusses with donor's relatives or legal representative to give information about the tissue procurement process and decide if donation was in accordance with the person's wishes, values and beliefs and whether the deceased had expressed an objection to donation during their lifetime. For vulnerable deceased people, the French law allows tissue procurement provided that the legal representative gives his authorization[16]. Vulnerable people included children and people under guardianship because of mental illness. The dataset we worked on was provided by tissue banks and completely anonymous. The consent issue was addressed prior to this work.

### Study objectives

**Fig 1:**
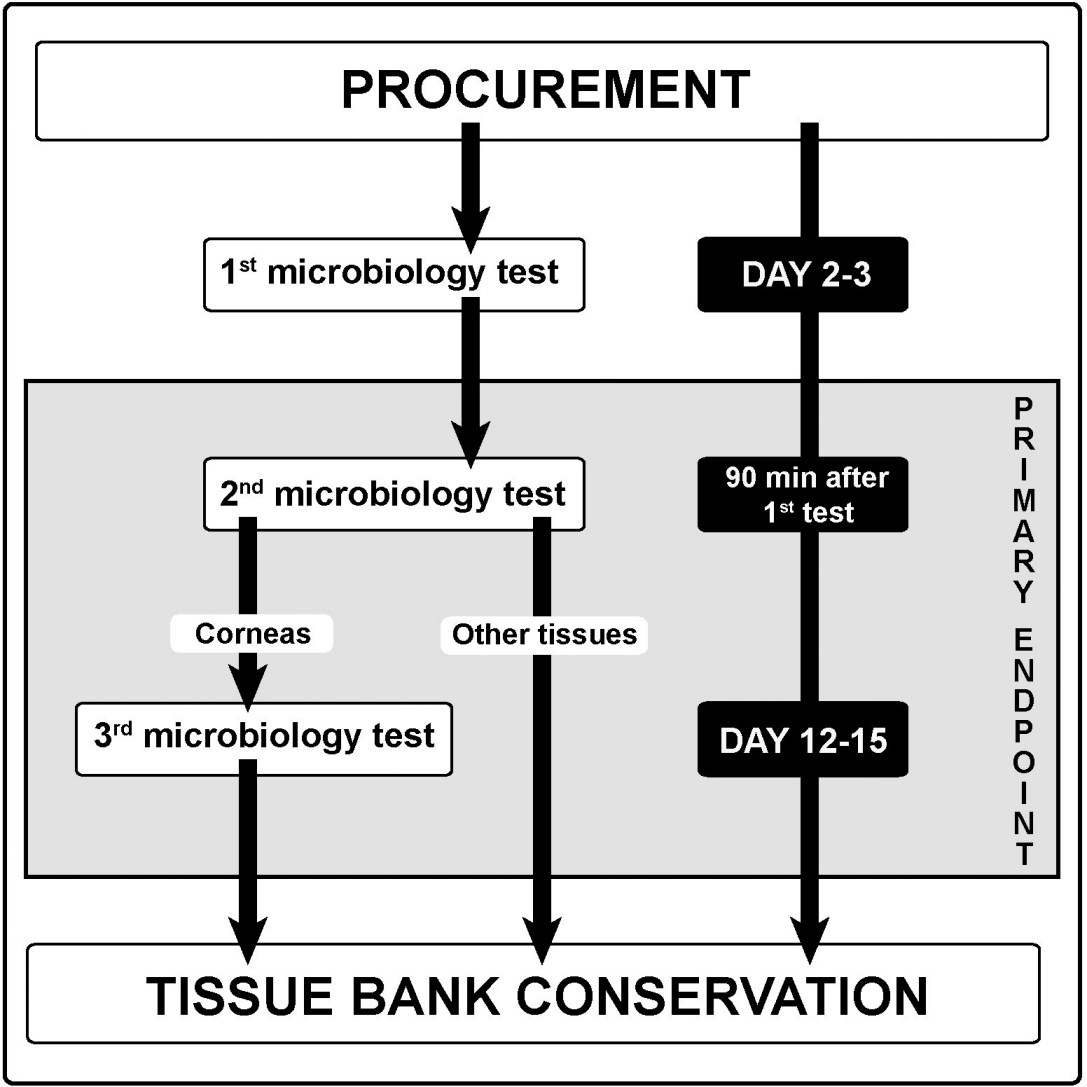
Summary of the tissue allograft microbiology contamination checking process. All positive results for the 2^nd^ or 3^rd^ tests defined tissue sample contaminations and were used as the primary endpoint of the present study.

The primary endpoint was the microbial contamination of the grafts (Fig 1). Our primary objective was to study the relationship between the risk of microbial contamination and the location (ICU room, SOR or NODR) of the tissue retrieval in heart-beating and non-heart-beating deceased donors. The secondary objective was to assess the contamination risk associated with other covariates (type of tissue, donor age, donor gender, duration of procurement process, cause and determination of the death). We also aimed to describe the microorganisms involved in the tissue grafts contamination.

### Techniques, data variables and definitions

All tissue retrieval procedures were performed according to EDQM guidelines, by trained personnel, operating under aseptic conditions after hand disinfection and wearing sterile clothes, sterile gloves and surgical facemasks[4]. The surgical techniques, conservation solutions, storage temperature and storage duration were the same for each hospital and for each tissue and are summarized in appendix in S1 Appendix. The microbiological protocols used to assess tissue contaminations are reported in appendix in S2 Appendix.

The contamination checking process routinely implemented at the tissue banks is summarized in Fig 1 and was used as the primary endpoint of the present study. Tissues processing, which require more stringent clean and air control than procurement, was separately performed by tissue establishments in an area class A of the GMP classification[17] and a background environment of Grade B to D as recommended (4). All tissues were considered for analysis except from bone tissues, as they are necessary procured in operating theatres, due to sterility requirements. Other data collected were: donor gender (male or female), donor age, location of the procurement process (ICU, SOR or NODR), death determination (brain death or circulatory death), cause of the death (gathered in 3 categories: cerebrovascular diseases, trauma and other), time between death and the start of the tissue removal, duration of the procurement process, failure to pass the quality and safety process, microbial agents involved in contamination. Reasons for failing the safety and quality process were: microbial contamination, abnormal cells count for corneal tissues, positive viral serologic tests and other reasons. Cerebrovascular diseases included stroke and cerebral hemorrhage. Among other causes of death we found anoxic cerebral injuries following cardiac arrest. The start of the procurement was defined as the start of the surgical removing of the tissue and the end as the storing of the graft in the preservation solution. The delay between these two times defined the duration of the procurement. Although the present study consisted in a retrospective analysis, all data were prospectively collected, as it was a part of the usual tissue banking process.

### Statistical analysis

Descriptive statistics were reported using mean and standard deviation for continuous variables. For categorical variables, frequencies and proportions were given. To study the influence of the procurement location on microbial contamination we used a multivariate regression model involving 8 covariates (3 continuous and 5 categorical variables): cause of death, location of the procurement process, donor age, donor gender, death determination (brain or circulatory death), type of tissue, time between death and the start of the procurement, duration of the procurement process. Because several observations could be related to the same donor, we needed a model allowing us to account for correlation between grafts provided by the same donor. For this purpose, we chose a GEE (generalized estimating equations) model with an exchangeable correlation structure. This method allows parameters of generalized linear model to be estimated in clustered data[18] and returns a marginal model that fits in with our purpose (i.e. estimating average response in overall population)[19]. We managed continuous covariates by assuming a linear relation with the outcome. In order to consider nonlinearity issues, we also planned to check if estimates given by a model handling continuous covariates with a restricted cubic splines approach[20] were not significantly different. 95% confidence intervals (CI) for odds ratio (OR) were given in the univariate and the multivariate analysis based on the robust standard error provided by the Huber-White standard error[21] and assuming the normal distribution of the regression coefficients. For the multivariate analysis we also calculated the 95% CI with a non-parametric bootstrap approach consisting in resampling 1000 times on the cluster level[22] with no replacement. To test the significance of OR we performed Wald tests. A P value of 0.05 was considered to indicate statistical significance. Because it was a clustered study with few observations per cluster, we aimed at collecting a large amount of donors (> 1000) to ensure accuracy of coefficients estimates[23]. To achieve this target we queried the tissue bank database from 2007 to 2014 allowing us to collect at least 2000 observations. All statistical analysis were performed using R software (version 3.3.2)[24].

## Results

### Studied population characteristics

From January 2007 to December 2014, 2800 observations were collected of which 1 was removed because the outcome was missing, 80 because related to bone tissues and 184 because of too much missing values for covariates. Finally, 2535 observations were analyzed from 1027 donors consisting in 658 skin grafts, 1525 corneal grafts, 46 heart valves grafts and 306 blood vessels grafts. Characteristics of the population are reported in the Table 1. 1394 (55%) procurements followed a circulatory death whereas 1141 (45%) followed a brain death. The retrieval took place for 1189 (47%) in a SOR, for 996 (39%) in a hospital mortuary (NODR) and for 350 (14%) in an ICU.

**Table 1:** Population characteristics.

Variable	Overall n = 2535	Contamination n = 285	No contamination n = 2250
Location			
Operating room	1189 (47%)	128 (11%)	1061 (89%)
Hospital mortuary	996 (39%)	87 (9%)	909 (91%)
ICU	350 (14%)	70 (20%)	280 (80%)
Death			
Circulatory death	1394 (55%)	167 (12%)	1227 (88%)
Brain death	1141 (45%)	118 (10%)	1023 (90%)
Gender			
Female	858 (34%)	73 (9%)	785 (91%)
Male	1677 (66%)	212 (13%)	1465 (87%)
Cause of death			
Other	994 (39%)	117 (12%)	877 (88%)
CVD	678 (27%)	59 (9%)	619 (91%)
Trauma	863 (34%)	109 (13%)	754 (87%)
Type of tissue			
Corneas	1525 (60%)	114 (7%)	1411 (93%)
Skin	658 (26%)	131 (20%)	527 (80%)
Blood vessels	306 (12%)	36 (12%)	270 (88%)
Heart valves	46 (2%)	4 (9%)	42 (91%)
Age (years)	57 ± 17	55 ± 18	57 ± 17
Time since death (h)	11.7 ± 7.5	11.9 ± 9.6	11.7 ± 7.2
Duration (min)	39 ± 30	47 ± 31	38 ± 29

For categorical variables, results are given as number of patients (percentage), for continuous variables as mean ± SD. ICU = Intensive Care Unit, SD = standard deviation, CVD = Cerebrovascular disease, h = hours, min = minutes.

### Grafts characteristics

Over the 2535 available grafts, 285 (11%) microbial contaminations were revealed. It concerned respectively 131 (20%), 114 (7%), 4 (9%) and 36 (12%) from skin, corneal, heart valves and arterial grafts. 57 different species of micro organisms were involved, 52 bacteria accounting for 230 contaminations, and 9 fungi responsible for 41 contaminations (Tables 2 and 3). In 15 cases no organism was specified. Over the 230 bacterial contaminations, 128 (56%) were due to *Staphylococcus* species. Candida accounted for 21 of the 41 fungi contaminations (52%). 355 grafts failed to pass the safety and quality process, 244 because of microbial contamination, 42 for cell count, 30 because of positive viral serologic test and 39 for unspecified reasons. Over the 285 contaminations only 244 didn't pass the process. First, for some contaminations, especially due du coagulase-negative staphylococci, transplantation is not necessarily a contraindication. Second, a first testing could show a contamination whereas a second testing after an antibiotic course could be negative leading to accept the tissue for transplantation.

**Table 2:** Number of microbial contaminations for each kind of tissue and microorganisms involved.

	Corneas	Skin	Heart valves	Blood vessels	Total
**TOTAL**	**114 (100%)**	**131 (100%)**	**4 (100%)**	**36 (100%)**	**285 (100%)**
**Micro organism**					
**Gram-positive bacteria** ***Staphyloccus aureus*** **Non aureus *staphylococci*** ***Streptococci*** ***Enterococci***	**30 (26.3%)** 0 24 (21%) 0 1 (0.9%)	**92 (70.2%)** 5 (3.8%) 72 (55%) 0 8 (6.1%)	**2 (50%)** 0 2 (50%) 0 0	**28 (77.8%)** 0 25 (69.4%) 1 (2.8%) 0	**152 (53.3%)** 5 (1.7%) 123 (43.2%) 1 (0.3%) 9 (3.2%)
**Gram-negative bacteria** ***Enterobacter*** ***Pseudomonas aeruginosa*** ***Escherichia coli***	**43 (37.7%)** 1 (0.9%) 6 (5.3%) 7 (6.1%)	**24 (18.3%)** 11(8.4%) 3(2.3%) 2(1.5%)	**0** 0 0 0	**7 (19.4%)** 1(2.8%) 0 1(2.8%)	**74 (26%)** 13 (4.6%) 9 (3.2%) 10 (3.5%)
***Anaerobes***	1 (0.9%)	3(2.3%)	0	3(8.3%)	7 (2.5%)
**Fungi** ***Candida***	**26 (22.8%)** 16 (14%)	**14 (10.7%)** 5 (3.8%)	**0** 0	**1 (2.8%)** 0	**41 (14.4%)** 21 (7.4%)
**Mycobacteria**	**3 (2.6%)**	**0**	**0**	**0**	**3 (1%)**
**Unspecified**	**12 (10.5%)**	**1 (0.8%)**	**2 (50%)**	**0**	**15 (5.3%)**

**Table 3:** Microorganisms involved in microbial contamination considering each location.

	SOR	NODR	ICU
**TOTAL**	**128 (100%)**	**87 (100%)**	**70 (100%)**
**Micro organism**			
**Gram-positive bacteria:** ***Staphyloccus aureus*** **Non aureus *staphylococci*** ***Streptococci*** ***Enterococci***	**84 (65.6%)** 5 (4%) 71 (55.5%) 0 4 (3%)	**23 (26.4%)** 0 19 (21.8%) 1 (1.1%) 1 (1.1%)	**45 (64.3%)** 0 33 (47.1%) 0 4 (5.7%)
**Gram-negative bacteria:** ***Enterobacter*** ***Pseudomonas aeruginosa*** ***Escherichia coli***	**23 (17.2%)** 3 (2.3%) 2 (1.6%) 3 (2.3%)	**34 (39%)** 1 (1.1%) 5 (5.7%) 4 (4.6%)	**17 (24.3%)** 9 (12.8%) 2 (2.8%) 3 (4.3%)
**Anaerobes bacteria**	5 (4%)	2 (2.3%)	0
**Fungi:** ***Candida***	**19 (14.8%)** 7 (5.5%)	**18 (20.7%)** 3 (3.4%)	**4 (5.7%)** 11 (15.7%)
**Mycobacteria**	**0**	**1 (1.1%)**	**2 (2.8%)**
**Unspecified**	**2 (1.6%)**	**11 (12.6%)**	**2 (2.8%)**

SOR = Standard Operating room, NODR = Non-Operating Dedicated Room i.e. hospital mortuary, ICU = Intensive Care Unit.

### Multivariate analysis

**Fig 2:**
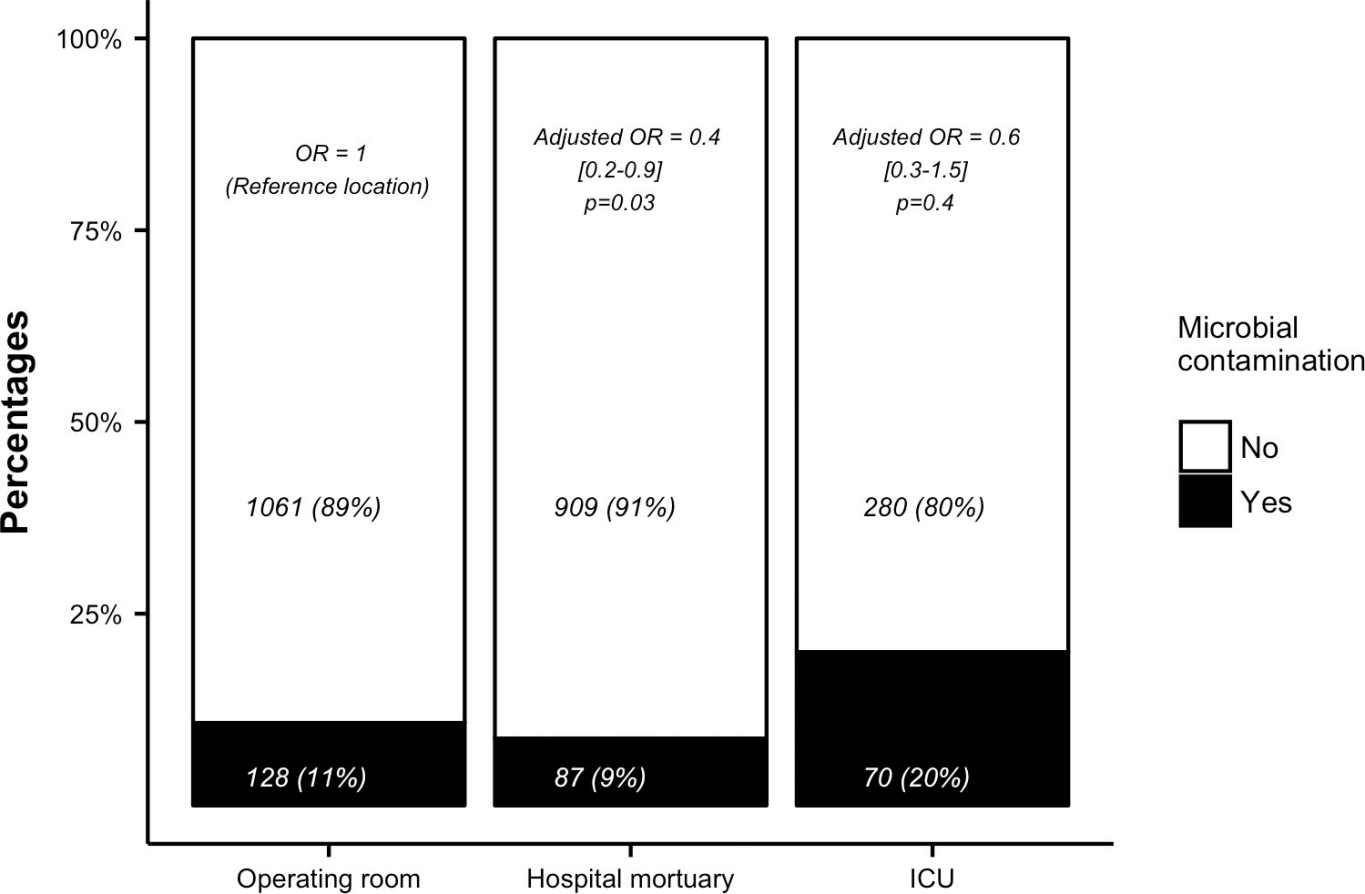
Microbial contamination according to the location of procurement. For each location, number of contaminated and uncontaminated grafts (percentages) with corresponding adjusted odds ratio (OR) are given.

**Fig 3:**
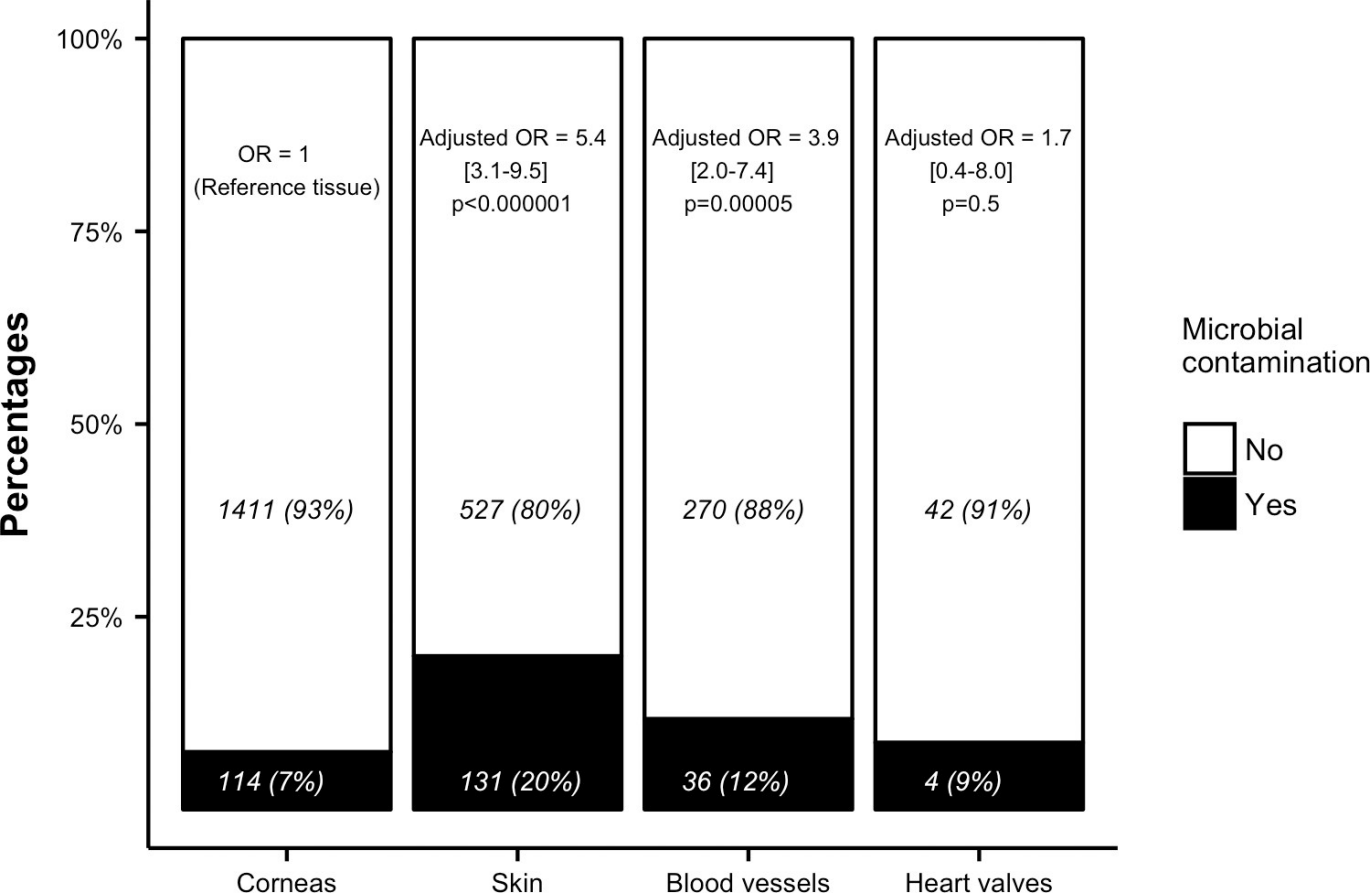
Microbial contamination according to the type of tissue. For each tissue, number of contaminated and uncontaminated grafts (percentages) with corresponding adjusted odds ratio (OR) are given.

The multivariate analysis, reported in Table 4, found 4 variables significantly associated with an increased risk of contamination: age expressed in year (Odd Ratio (OR) 1.02, 95% CI [1.001–1.03], p = 0.03), skin tissues (OR 5.42, 95% CI [3.09–9.52], p = <0.001), blood vessels tissues (OR 3.86, 95% CI [2.01–7.43], p<0.001) and time between death and start of the procurement process expressed in hour (OR 1.04, 95% CI [1.01–1.07], p = 0.01). Two variables were associated with a lower risk of infection: hospital mortuary rather than SOR (OR 0.43, 95% CI [0.2–0.91], p = 0.03) and brain death rather than circulatory arrest (OR 0.16, 95% CI [0.06–0.4], p<0.001). The multivariate analysis was unable to show an increased contamination risk when the procurement was performed in the ICU (OR 0.62, 95% CI [0.26–1.48], p = 0.4). Using a restricted cubic splines to handle nonlinearity of continuous predictors, we found no difference in estimated coefficients comparing with a linear approach[20]. Fig 2 displays the number of grafts procured in the 3 different locations, the respective number of contaminations and corresponding OR. Fig 3 shows the percentage of contamination associated with each kind of tissue and the corresponding OR.

**Table 4:** Univariate and multivariate analyses.

		Univariate analysis		Multivariate analysis		
Variable		OR [95% CI]	P value	Adjusted OR [95% CI]	Bootstrap 95% CI	P value
**Location**	Operating room	1		1		
	Hospital mortuary	0.9 [0.6–1.4]	0.8	0.4 [0.2–0.9]	[0.2–0.9]	0.03
	Intensive care unit	2.7 [1.5–4.7]	<0.001	0.6 [0.3–1.5]	[0.2–1.5]	0.4
**Death**	Circulatory arrest	1		1		
	Brain death	0.7 [0.5–1.02]	0.06	0.2[0.1–0.4]	[0.1–0.4]	<0.001
**Age (year)**		1 [0.98–1.06]	0.5	1.02 [1.001–1.03]	[1–1.03]	0.03
**Gender**	Male	1		1		
	Female	1.1 [0.8–1.6]	0.5	1.2 [0.8–1.8]	[0.8–1.7]	0.4
**Cause of death**	CVD	1		1		
	Other	1.6 [1.01–2.5]	0.046	1.4 [0.8–2.4]	[0.8–2.3]	0.2
	Trauma	1.4 [0.9–2.3]	0.2	1.6 [0.9–2.8]	[0.9–2.8]	0.1
**Tissue**	Corneas	1		1		
	Skin	3.6 [2.4–5.3]	<0.001	5.4 [3.1–9.5]	[3–10]	<0.001
	Blood vessels	2 [1.3–3]	<0.001	3.9 [2–7.4]	[2–8]	<0.001
	Heart valves	1.2 [0.3–4.5]	0.8	1.7 [0.4–8]	[0.3–9]	0.5
**Time since death (hours)**		0.99 [0.96–1.03]	0.9	1.04 [1.01–1.07]	[1.02–1.1]	0.01
**Duration (minutes)**		1.01 [1–1.02]	<0.001	1 [0.99–1.01]	[0.99–1.01]	0.7

For categorical covariates, the first level is the reference with a corresponding OR of 1. OR were calculated with a GEE model assuming an exchangeable correlation structure and involving only one explanatory variable for the univariate analysis and the whole set of variables for the multivariate analysis. P values result of a Wald test. Bootstrap CI were built by resampling 1000 times on the cluster level (donor) with replacement and calculated as follow: estimated OR of the initial multivariate model ± SD estimated from the 1000 bootstrap samples. OR = Odds ratio, CI = Confidence interval, GEE = Generalized Estimating Equations, CVD = Cerebrovascular disease.

## Discussion

Theoretically, SOR with trained personnel and air-quality control represent the best place for procurement. The present study challenges this common idea showing that mortuary rooms (NODR) were associated with a lower risk of tissue microbial contamination as compared to SOR (Fig 2). Moreover, skin and blood vessels tissues were at higher risk of contamination, as compared with cornea or cardiac valves (Table 3, Fig 3).

The influence of the site of tissue procurement has been poorly studied. To the best of our knowledge, this is the first report describing tissue recovery in ICU rooms. In USA, tissue recovery can be done in SOR, NODR, autopsy room or funerals home[25]. In a study analyzing all tissue samples from 1031 donors recovered in 7 tissue banks, SOR was identified as a protective factor for tissue contamination. In this study, positive cultures were 5 to 11% lower for the SOR versus the others locations, making it a small factor affecting the contamination risk. Authors suggested that surgical technique, site preparation, site disinfection and surgical isolation techniques play a larger role in preventing contamination than the recovery environment[25]. In this study, recoveries after autopsy, length of time taken for recovery, recovery teams with less than 3 members and skin recovery were important factors for tissue contamination[25]. For European guidelines SOR is not mandatory, even if for cardiovascular tissues it is recommended[4]. These guidelines mention that the environment for tissue retrieval should have the following characteristics:

- Adequate size regarding floor space and work-tops that will be used,
- Appropriately located to ensure cleanliness and privacy,
- Sufficient and suitable lighting,
- Good state of repair,
- Free of pests,
- Sufficiently clean or cleanable not to contribute to cells or tissues contamination.

These characteristics suggest that a room not monitored for air quality is sufficient for tissue recovery. This can be applied to SOR, NODR or ICU rooms and explains why tissue procurement is done in these 3 settings. They also recommend assessing contamination risk to choose the kind of facility used for retrieval, but no reproducible and clear rule is provided. Our results suggest that non-operating dedicated rooms (NODR) are at lower risk of contamination as compared to SOR. Several explanations could be advanced. First, in the present study, tissue recovery performed in SOR was exclusively done in brain-dead donors after multiple organ recovery. In such case, the time from cardiac arrest to tissue recovery cannot be standardized. In case of rapid procedure (only kidneys recovery), tissue retrieval is performed less than 6 hours after cardiac arrest. In case of multiple organ recovery: heart, lungs, liver, pancreas, kidneys, the time from cardiac arrest to tissue recovery can be longer than 6 hours. European guidelines emphasized the fact that tissue recovery should be done as earlier as possible, in the 24 hours following cardiac death, if a body cooling can be done within the 6 hours following death[4]. In case of multiple organ retrieval, this time interval cannot be reached.

Second, prolonged body cooling has been proposed as a protective factor for microbial contamination[6, 25]. In case of recovery in NODR, body cooling was systematically performed for 8 to 12 hours. This prolonged cooling can decrease bacterial growth and therefore bacterial inoculum at the time of recovery. However, our results do not allow confirming this hypothesis.

Third, cornea samples were mainly recovered in NODR, while skin and cardiac tissues retrieval were mainly performed in SOR or ICU. As report in Fig 3, skin and vascular tissues are associated with a greater risk of contamination. A multivariate analysis could imperfectly adjust for measured confounders. Thus, a selection bias due to a greater proportion of cornea recovery in NODR could explain the difference of contamination observed in the present report.

Fourth, the temperature in the hospital mortuary is lower than in the operating room. Because hypothermia in the operating room is commonly described as responsible for significant morbidity, a not too low temperature is advocated. In France, standards mandate a mean temperature between 19°C and 26°C[26]. Such recommendations are found worldwide[27]. On the contrary, for hospital mortuary, a temperature below 17°C is recommended in France[28]. It could be expected that a higher temperature in the operating room could lead to an increased risk of tissue infection.

Finally, because it was an observational study, issues due to unknown confounders were not handled. Especially, residual antibiotics concentrations can play a role in tissue contamination[29, 30]. Tissue banks use a wide variety of homemade antibiotics cocktails in order to limit microbial tissue allograft contamination and to maximize the safety of allografts[31]. Buzzi et al recently demonstrated that antibiotic residues present in cardiac tissue allografts and processing liquids after decontamination may mask microbial contamination during microbiological analysis performed with standard tissue bank methods, leading to false negatives[32]. Some studies suggest that the use of specific devices (RESEP) allowing antibiotics residues removal from liquid samples can help to limit false negative[32]. For tissue procurement procedure performed in SOR or ICU, it can be hypothesized that donors have previously received broad-spectrum systemic antibiotics for infection. These antibiotics can increase the risk of residual antibiotics concentrations and lead to false negative allograft testing performed just before graft.

### Study limitations

The retrospective design of the study cannot allow a definitive conclusion. Further prospective, comparative studies are needed to elucidate this issue. More, some contamination factors couldn't be studied to further understand the contamination process in procurement. Especially, bacterial environment evaluation and air quality characteristics were not available. Because we studied more than 1000 patients and more than 2500 allografts, we think that our study plea for comparative studies.

## Conclusion

According to our results, performing tissue procurement in non-operating dedicated rooms, when no organ retrieval is needed, could decrease the rate of allograft tissue contamination. This study also suggests that in daily clinical practice, transferring patients from ICU to SOR for tissue procurement could be avoided as it does not lead to less microbial contamination. ICU seems to be a valuable alternative for tissue retrieval.

## Supporting information

S1 Appendix Procurement procedures. (DOCX)

S2 Appendix Microbiological protocols used to assess tissue contaminations. (DOCX)
