# Supplementary material for: Microbial contamination and tissue procurement location: A conventional operating room is not mandatory. An observational study
Source: PLoS One. 2019 Jan 8;14(1):e0210140. doi: 10.1371/journal.pone.0210140 (PMC6324794; doi:10.1371/journal.pone.0210140)
Supplement: S1 Appendix — (DOCX) [file pone.0210140.s001.docx]

**S1 Appendix. Procurement procedures.**

1- Pre-operative procedure

- Shaving of the donor areas when necessary, with electrical clippers
- Disinfection with Betadine® Scrub (7.5% povidone-iodine) and scrubing thoroughly for 2 minutes
- Rinsing off the skin with sterile water.
- Applying povidone-iodine 10%, two times
- Remaining the solution wet on the skin for at least 2 minutes

2- Preparation of the procurement team

- Surgical facemask, sterile clothes and sterile gloves
- Surgical hand washing
- Sterile field on surgical table and surgical site

3- Tissue retrieval procedures

- Corneas:
  - Excision in situ carrying the cornea and a scleral collar with an overall diameter of at least 17 mm
  - Storage in a medium compatible with the shelf life: CORNEA MAX® (Lab. Eurobio, France) at ambient temperature
- Skin:
  - Battery-operated dermatome adjusted between 200 and 400 microns
  - Skin grafts cut from the lower limbs
  - A new blade is used for each area
  - Storage in 250 ml of an organ culture medium cooled to +4 ° C
  - Storage temperature between +2 and +8 ° C
- Heart valves:
  - Cannulation of the ascending aorta as high as possible. The explanted core disposed in a sterile package, in the culture medium used for the preservation of the valves before cryopreservation
  - Storage temperature +4 ° C
- Blood vessels:
  - All large and medium-sized arterial segments present in the surgical field after any organ removal could be taken.
  - Storage temperature between +2 and +8°C

4- Conservation solutions

- Cornea: CORNEA MAX® Solution, Eurobio Lab, France.
- Skin: Solution SCOT 30® + cefamandole (235μg / ml), dalacin (128μg / ml), colimycin (742UI / ml), vancomycin (100μg / ml), fungizone (50μg / ml) and gentamicin (320μg /ml)
- Heart valves: Solution SCOT 30®, Macopharma Lab, France without antibiotic.
- Blood vessels: SCOT 30® solution + cefamandole (235μg / ml), dalacin (128μg / ml), colimycin (742UI / ml), vancomycin (100μg / ml).
