# Supplementary material for: Microbial contamination and tissue procurement location: A conventional operating room is not mandatory. An observational study
Source: PLoS One. 2019 Jan 8;14(1):e0210140. doi: 10.1371/journal.pone.0210140 (PMC6324794; doi:10.1371/journal.pone.0210140)
Supplement: S2 Appendix — (DOCX) [file pone.0210140.s002.docx]

**S2 Appendix**. **Microbiological protocols used to assess tissue contaminations.**

1- Corneas

Corneas are tested for microbiological safety after at least 7 days in a 100 ml organ culture medium (CorneaMax^®^, Eurobio, France), one donor cornea in one Cornea Max^®^ culture medium. Two culture bottles, one for aerobic testing (BACT/ALERT^®^ FA Plus, Biomerieux, France) and one for anaerobic testing (BACT/ALERT^®^ SN Plus, Biomerieux, France), are seeded, each with 5 ml of culture medium. In addition, one Sabouraud liquid medium (SAB B-T^®^, Biomerieux, France) and one Brain Heart Infusion medium (BBL^TM^ Brain Heart Infusion, Becton-Dickinson, USA) are seeded with 1 ml of culture medium. Blood culture vials are incubated in an automated culture system for 10 days. Brain Heart infusion and Sabouraud medium are incubated respectively at 37°C and 30°C for 10 days.

2- Skin grafts

Skin patches are tested after 24h to 48h at +2 to +8°C in a 250 ml solution SCOT30® (SCOT30®, Macopharma, France) with antibiotics (cefamandole, 235 µg/ml; clindamycin, 128 µg/ml; colistine, 742 UI/ml; vancomycin, 100 µg/ml; amphotericin B, 50 μg/ml; gentamicin, 320 µg/ml). This solution is also the procurement medium. At the tissue bank, a first bacteriological sampling is prepared in a laminar flow cabinet (GMP class A) in a class B clean room. Two culture bottles, one for aerobic and one for anaerobic testing are seeded, each with 5 ml of the procurement medium. In addition, one Schaedler K3® liquid medium (SCHAEDK3^®^ 0.2%-T, Biomerieux, France) and one Tryptone Soya Broth medium (USP, Oxoid^TM^, USA) are seeded with 1 ml of the procurement medium. Then, skin patches are processed for cryopreservation under sterile conditions. At the end of the processing, the cryopreservation medium (SCOT30® + 10% DMSO (Dimethyl sulfoxid) + antibiotics as before) soaking skin patches is sampled in two blood cultures bottles (5 ml in each vial), aerobic and anaerobic. Blood culture vials are incubated in an automated culture system for 10 days. Schaedler K3® and Tryptone Soya Broth are incubated at 37°C for 10 days.

3- Vascular tissues

Vascular tissues are tested for microbiological safety after 24h to 48h at +2/+8°C in 250 ml of a SCOT30® solution (SCOT30®, Macopharma, France) with antibiotics (cefamandole, 235 µg/ml; clindamycin, 128 µg/ml; colistine, 742 UI/ml; vancomycin, 100 µg/ml). At the tissue bank, a first bacteriological sampling is prepared in a laminar flow cabinet (GMP class A) in a class B clean room. Two blood culture vials, one for aerobic testing and one for anaerobic testing, are seeded, each with 5 ml of the procurement medium. In addition, one Schaedler K3® liquid medium (SCHAEDK3® 0.2%-T, Biomerieux, France) is seeded with 1 ml of the procurement medium. Then, vascular tissues are processed for cryopreservation under sterile conditions. At the end of the processing, the cryopreservation medium (SCOT 30® + 10% DMSO, without antibiotics) soaking vascular tissues is sampled (5 ml in each vial) in two blood cultures bottles, aerobic and anaerobic and 1ml in a Schaedler K3® medium. Blood culture vials are incubated in the automated blood culture system for 10 days. Schaedler K3 is incubated at 37°C for 10 days.
